# Association of Early Repolarization Pattern on ECG with Risk of Cardiac and All-Cause Mortality: A Population-Based Prospective Cohort Study (MONICA/KORA)

**DOI:** 10.1371/journal.pmed.1000314

**Published:** 2010-07-27

**Authors:** Moritz F. Sinner, Wibke Reinhard, Martina Müller, Britt-Maria Beckmann, Eimo Martens, Siegfried Perz, Arne Pfeufer, Janina Winogradow, Klaus Stark, Christa Meisinger, H.-Erich Wichmann, Annette Peters, Günter A. J. Riegger, Gerhard Steinbeck, Christian Hengstenberg, Stefan Kääb

**Affiliations:** 1 University Hospital Munich, Campus Grosshadern, Medical Department I, Ludwig-Maximilians University Munich, Munich, Germany; 2 Klinik und Poliklinik für Innere Medizin II, Universitätsklinikum Regensburg, Regensburg, Germany; 3 Institute of Epidemiology, Helmholtz Zentrum München, Neuherberg, Germany; 4 Institute of Biological and Medical Imaging, Helmholtz Zentrum München, Neuherberg, Germany; 5 Institute of Human Genetics, Helmholtz Zentrum München, Neuherberg, Germany; 6 Institute of Human Genetics, Technical University Munich, Munich, Germany; 7 Institute of Medical Informatics, Biometry and Epidemiology, Chair of Epidemiology, Ludwig-Maximilians-Universität, Munich, Germany; 8 Klinikum Grosshadern, Munich, Germany; University of Bristol, United Kingdom

## Abstract

**Background:**

Early repolarization pattern (ERP) on electrocardiogram was associated with idiopathic ventricular fibrillation and sudden cardiac arrest in a case-control study and with cardiovascular mortality in a Finnish community-based sample. We sought to determine ERP prevalence and its association with cardiac and all-cause mortality in a large, prospective, population-based case-cohort study (Monitoring of Cardiovascular Diseases and Conditions [MONICA]/KORA [Cooperative Health Research in the Region of Augsburg]) comprised of individuals of Central-European descent.

**Methods and Findings:**

Electrocardiograms of 1,945 participants aged 35–74 y, representing a source population of 6,213 individuals, were analyzed applying a case-cohort design. Mean follow-up was 18.9 y. Cause of death was ascertained by the 9th revision of the International Classification of Disease (ICD-9) codes as documented in death certificates. ERP-attributable effects on mortality were determined by a weighted Cox proportional hazard model adjusted for covariables. Prevalence of ERP was 13.1% in our study. ERP was associated with cardiac and all-cause mortality, most pronounced in those of younger age and male sex; a clear ERP-age interaction was detected (*p* = 0.005). Age-stratified analyses showed hazard ratios (HRs) for cardiac mortality of 1.96 (95% confidence interval [CI] 1.05–3.68, *p* = 0.035) for both sexes and 2.65 (95% CI 1.21–5.83, *p* = 0.015) for men between 35–54 y. An inferior localization of ERP further increased ERP-attributable cardiac mortality to HRs of 3.15 (95% CI 1.58–6.28, *p* = 0.001) for both sexes and to 4.27 (95% CI 1.90–9.61, *p*<0.001) for men between 35–54 y. HRs for all-cause mortality were weaker but reached significance.

**Conclusions:**

We found a high prevalence of ERP in our population-based cohort of middle-aged individuals. ERP was associated with about a 2- to 4-fold increased risk of cardiac mortality in individuals between 35 and 54 y. An inferior localization of ERP was associated with a particularly increased risk.

*Please see later in the article for the Editors' Summary*

## Abstract 2

In a population-based cohort study of middle-aged people in Central Europe, Stefan Kääb and colleagues find an association between electrocardiographic early repolarization pattern and mortality risk.

## Editors' Summary

**Background:**

Cardiovascular diseases—disorders that affect the heart and the circulation—are the leading cause of death in the developed world. About half of cardiovascular deaths occur when the heart suddenly stops pumping (sudden cardiac arrest). The muscular walls of the four heart chambers contract in a set pattern to pump blood around the body. The heart's internal electrical system controls the rate and rhythm of these contractions and, if this system goes wrong, an abnormal heart beat or “arrhythmia” develops. Some arrhythmias—in particular, ventricular fibrillation in which the walls of the two lower heart chambers quiver or “fibrillate” instead of pumping—can cause sudden cardiac arrest and immediate loss of consciousness. Death follows within minutes in 95% of cases but immediate cardiopulmonary resuscitation (CPR; chest compression to pump the heart and inflation of the lungs by mouth-to-mouth resuscitation) can keep a person alive until a defibrillator can be used to restore the normal heart beat. People who survive sudden cardiac arrest can be given anti-arrhythmia drugs or have a pacemaker implanted to stabilize their heart beat.

**Why Was This Study Done?:**

The beating heart generates tiny electric waves that can be detected by electrodes on the skin. The pattern of these waves (an electrocardiogram or ECG) provides information about the heart's health. One wave pattern that is often seen on ECGs is the “early repolarization pattern” (ERP), which some studies suggest is associated with an increased risk of cardiac death. Here, the researchers investigate the prevalence of ERP (the proportion of a population with ERP) and its association with death from heart-related problems (cardiac mortality) and from any cause (all-cause mortality) in the MONICA/KORA prospective, population-based case-cohort study. The MONICA Project (MONitoring of Trends and Determinants in CArdiovascular Disease) has studied cardiovascular disease in 10 million people in 21 countries; KORA denotes the study done in the Augsburg region of Germany. In a prospective study, specific baseline characteristics of the study's participants are determined and the participants are followed to see who experiences a predefined outcome. A case-cohort study investigates a randomly selected subcohort (subgroup) of the original participants of a study and any participants who experience the predefined outcome instead of all the participants.

**What Did the Researchers Do and Find?:**

The researchers selected 1945 MONIKA/KORA participants aged 35–74 years from a source population of about 6,000 people using a case-cohort study design. They analyzed the ECGs (recorded in 1984–1985 or 1989–1990) of this subcohort and ascertained the cause of death for those participants who died during the 18.9 year average follow-up. The overall prevalence of ERP in the study was 13.1%, report the researchers, and ERP was associated with cardiac mortality, particularly among younger and male participants. Specifically, among men and women aged 35–54 years, having ERP was associated with a nearly doubled risk of cardiac death. Among men aged 35–54 years, having ERP was associated with an increase in the risk of cardiac death by 2.65-fold. An ERP localized to the bottom of the heart (inferior localization) was associated with an increased risk of cardiac death among both sexes by more than 3-fold and among men by more than 4-fold in this age group. Finally, ERP was also significantly associated with an increased risk of all-cause mortality but less strongly than with cardiac mortality.

**What Do These Findings Mean?:**

These findings suggest that the prevalence of ERP among the middle-aged people in the MONICA/KORA study is high (and somewhat higher than previously reported). They also show a clear association between ERP and the risk of cardiac death among 35–54-year-old people, particularly among men, but because of the study design, these findings do not show that ERP actually causes cardiac death; it could simply be a susceptibility marker. The researchers note that the increased risk of cardiac death associated with ERP is of a similar size to that associated with some other ECG abnormalities. However, although it might be worth paying special attention to young people with an inferior localization of ERP, finding ERP in a person without symptoms and without a family history of sudden cardiac death should not lead to further investigations or any preventative therapy, they suggest, because the absolute risk of cardiac arrest in such people is very low.

**Additional Information:**

Please access these Web sites via the online version of this summary at http://dx.doi.org/10.1371/journal.pmed.1000314.

- The US National Heart Lung and Blood Institute provides information on cardiovascular conditions, including sudden cardiac arrest and on arrhythmias
- The American Heart Association also information on sudden cardiac death and on arrhythmias
- The German Cardiac Society (Deutsche Gesellschaft fr Kardiologie) and the German Heart Foundation (Deutsche Herzstiftung) provide further information (in German) on cardiovascular conditions
- The Heart Rhythm Foundation provides information on all aspects of heart arrhythmia
- The Fondation Leducq Alliance Against Sudden Cardiac Death provides information on sudden cardiac arrest
- MedlinePlus provides links to other resources about cardiac arrest and arrhythmias (in English and Spanish)
- The MedlinePlus Encyclopedia has a page on electrocardiograms (in English and Spanish)
- The Nobel Foundation provides an interactive electrocardiogram game
- More information about the MONICA project and the KORA Study or is available

## Introduction

The electrocardiographic pattern of early repolarization (ERP), a slurring or notching at the QRS-ST junction, is a common and long described finding on electrocardiogram (ECG) [1],[2]. Its prevalence has been assessed controversially, ranging between 1%–5%, and being more common in young athletic males [3]–[5]. Although generally considered benign, experimental studies suggested an arrhythmogenic potential [6], and recent clinical data demonstrated a strong association with ventricular fibrillation and sudden cardiac death (SCD) in patients without structural heart disease [7]. Recently, a community-based investigation of Finnish individuals demonstrated a slightly increased cardiovascular mortality associated with ERP [8].

Cardiovascular diseases are the main cause of death in the industrialized world, and in approximately 50% of deaths due to cardiovascular causes, SCD, mostly due to ventricular arrhythmias, is considered the underlying reason [9],[10]. Incidence rates range between 50–100 per 100,000 residents depending on definition and ascertainment of SCD in the investigated population.

Here we sought to determine the prevalence of ERP in a large population-based cohort (Monitoring of Cardiovascular Diseases and Conditions [MONICA]/KORA [Cooperative Health Research in the Region of Augsburg] study) of individuals of Central-European descent in the age range between 35–74 y and to investigate its potential association with cardiac and all-cause mortality using follow-up information of more than two decades. In addition, we performed age- and sex-stratified analyses.

## Methods

### Study Population and Mortality Follow-Up

We performed a prospective case-cohort study on the basis of the World Health Organization's population-based German MONICA study in Augsburg. We investigated the two independent, cross-sectional surveys S1 (1984–1985) and S2 (1989–1990) that were performed in the region of Augsburg, Southern Germany, and comprised 8,962 participants (4,505 men; 4,457 women) [11],[12]. Because cardiovascular mortality is low at younger ages, analysis was restricted to individuals aged 35–74 y, yielding a source population of 6,213 individuals (3,035 men; 3,178 women).

Following the case-cohort design [13],[14], a representative random sample of the entire source population was drawn (referred to below as the subcohort), stratified by sex and survey as described elsewhere [15]–[17]. It was enriched with all participants who died during follow-up. Primary analysis focused on cardiac mortality. Individuals were excluded if no electrocardiogram (ECG) was available (*n* = 95). We further excluded individuals with significant ventricular conduction delay (QRS>120 ms, *n* = 94), atrial fibrillation (*n* = 21), and implanted pacemaker (*n* = 4). Thereafter, the case-cohort population consisted of 1,945 individuals. This total included a random sample of 1,583 participants representative for the entire source population and, in addition, all those who died of cardiac (*n* = 511) or any causes (*n* = 1,496) during follow-up.

All individuals gave written informed consent to participation in the KORA study, approved by the Ethics Committee of the Bavarian Medical Association (Bayerische Ärztekammer) and the Bavarian commissioner for data protection and privacy (Bayerischer Datenschutzbeauftragter), and adhering to the principles outlined in the Declaration of Helsinki.

All participants were prospectively followed on a regular basis within the framework of KORA and follow-up information was available until December 2007 [12]. In case of death, death certificates were obtained from local health departments and evaluated using the 9th revision of the International Classification of Diseases (ICD-9). Death of cardiac causes was assumed for ICD-9 codes 390–429 and 798.

### ECG Recording and Definition of ERP

Study participants received 12-lead resting ECGs (Sicard 803 ECG system, Siemens), applying a standardized protocol after 10-min rest in a supine position. ECGs were recorded at the initial study visit. Routine ECG parameters were obtained from automated measurements.

ERP was assessed manually in all ECGs using paper prints. Quantitative measurements were performed by ruler-based visual inspection using Spacelab magnifying glasses for ECG diagnostics. The criteria for detection of ERP were exactly as recently described by Haïssaguerre and colleagues [7]. Briefly, classification as ERP required a J-point elevation ≥0.1 mV in ≥2 adjacent leads with either slurring or notching morphology. Leads V1 to V3 were not interpreted to avoid confusion with ECG patterns of Brugada-syndrome or right ventricular dysplasia. Presence or absence of ST-elevation in addition to J-point elevation was not taken into account and nonspecific intraventricular conduction delay was excluded from analysis.

Two trained cardiologists from different centers, blinded to clinical data and follow-up status, independently assessed all ECGs. In case of divergent results, a third blinded cardiologist reinterpreted the ECG, and a preliminary decision on ERP status was achieved by majority vote. There was a moderate to substantial strength of agreement between the two initial interpreters (κ = 0.521, proportion agreement 0.82). A total of 483 (23.4%) ECGs were judged by the third cardiologist. After preliminary decision on ERP status, two trained cardiologists jointly reassessed all ECGs that were considered ERP-positive, and a final decision on ERP status was reached by consensus.

### Statistical Analysis

Statistical analyses were performed with “R” (R Foundation for Statistical Computing), applying a case-cohort model using the robust variance estimation method, which is a weighted analysis design [13]. Sex- and survey-stratified sampling weights within the subcohort were: S1: men, 2.78, women, 4.03; S2: men, 4.20, women, 4.97.

Baseline characteristics were expressed as means or proportions as appropriate and compared between groups. The effect of ERP on covariables was tested by linear regression for continuous variables or logistic regression for dichotomous variables.

The relationship between ERP and mortality was calculated using a weighted Cox proportional hazards model [18], and visualized by Kaplan-Meier plots. All results were adjusted for covariables by multivariate analyses. Covariables included age, sex, and survey, or additionally a series of clinical characteristics. In case of age- and/or sex-stratified analyses, no further adjustment was performed for the respective variables. An interaction term (ERP×age) was included to determine age-dependent effects. With ERP coded as dummy variable and age recoded to reference age 35 y, this term indicates an additional change of the effect size with each year of age above 35, which only applies for participants with ERP. To better illustrate the age-dependent effect of ERP, we also performed age- and sex-stratified subgroup analyses with age groups defined as 35–54, 55–64, and 65–74 y.

Because of the long-term follow-up and the design of the study, violation of the proportional hazards assumption was likely to occur. Therefore, we allowed the baseline hazard in Cox proportional hazards regression to vary with age group, sex, survey, and smoking status. The latter was identified to have a strongly time-dependent effect on mortality using a test based on scaled Schoenfeld residuals [19]. For consistency, this adjustment was kept throughout all subgroup analysis. The QT interval was corrected according to the Framingham formula [20]. Statistical significance was considered for a two-sided *p*-value of <0.05.

## Results

After application of exclusion criteria, we present weighted results for the entire source population of 6,213 individuals from surveys S1 and S2 with a male proportion of 48.9% and a mean age of 52.0 y. Detailed clinical characteristics are depicted in Table 1. Discrepancies between ERP-positive and -negative individuals were detected for age, smoking status, diabetes mellitus, and high-density lipoprotein (HDL) cholesterol. There were no significant differences in clinical characteristics in those who died of cardiac causes compared to survivors. In individuals that died of any cause during follow-up, ERP-positive individuals included a higher proportion of smokers and had a lower prevalence of congestive heart failure.

**Table 1:** Baseline characteristics of the study population.

Study Population Characteristics	Entire Study Population (*n* = 6,213)				Deceased from Cardiac Causes (*n* = 511)				Deceased from any Cause (*n* = 1,496)			
	All	ERP+	ERP−	*p*-Value*	All	ERP+	ERP−	*p*-Value	All	ERP+	ERP−	*p*-Value*
**Male sex (%)**	3,035 (48.9%)	439 (54.1%)	2,596 (48.1%)	0.10	345 (67.5%)	60 (67.4%)	285 (67.5%)	0.98	932 (62.3%)	160 (65.6%)	772 (61.7%)	0.25
**Age (y)**	52.0±10.1	53.4±9.9	51.8±10.1	**0.03**	52.0±10.1	53.4±9.9	51.8±10.1	0.16	59.8±8.8	59.8±8.2	59.8±9.0	0.96
**Body mass index (kg/m^2^)**	27.1±4.1	27.38±4.0	27.0±4.1	0.25	27.1±4.1	27.4±4.0	27.0±4.1	0.71	28.1±4.3	28.2±4.2	28.0±4.3	0.51
**Total cholesterol/HDL ratio** a	4.6±2.3	4.9±2.0	4.5±2.3	**0.03**	5.6±3.4	5.8±2.6	5.5±3.5	0.57	5.2±3.1	5.4±2.2	5.2±3.2	0.45
**Arterial hypertension (%)** b	2,609 (41.2%)	378 (46.6%)	2,182 (40.4%)	0.14	345 (67.5%)	63 (70.8%)	282 (66.8%)	0.47	916 (61.3%)	152 (62.6%)	764 (61.1%)	0.66
**Nicotine abuse (%)** c	3,241 (52.2%)	476 (58.6%)	2,765 (51.2%)	**0.03**	343 (67.1%)	64 (71.9%)	279 (66.1%)	0.29	928 (62.0%)	167 (68.4%)	761 (60.8%)	**0.02**
**Congestive heart failure (%)** d	312 (5.0%)	21 (2.6%)	291 (5.38%)	0.06	70 (13.7%)	9 (10.1%)	61 (14.5%)	0.28	164 (11.0%)	16 (6.6%)	148 (11.8%)	**0.02**
**Prior myocardial infarction(%)** d	149 (2.4%)	13 (1.5%)	137 (2.5%)	0.53	55 (10.8%)	8 (9.0%)	47 (11.1%)	0.55	88 (5.9%)	13 (5.3%)	75 (6.0%)	0.69
**Diabetes mellitus (%)** d	256 (4.1%)	60 (7.4%)	196 (3.6%)	**0.04**	80 (15.7%)	10 (11.2%)	70 (16.6%)	0.21	168 (11.2%)	29 (11.9%)	139 (11.1%)	0.72
**Heart rate (min^−1^)**	66.3±10.9	65.81±10.6	66.3±11.0	0.52	68.4±12.5	69.7±14.3	68.2±12.1	0.31	68.5±12.1	68.2±12.2	68.6±12.0	0.70
**QTc (ms)^|^** e	398.4±19.7	396.5±17.9	398.7±20.0	0.14	402.2±24.4	400.4±20.4	402.6±25.2	0.45	401.3±21.4	399.6±21.2	401.6±21.5	0.19

Results for the study population are weighted using the respective sample weights of the case-cohort study. Continuous variables are expressed as mean ± standard deviation, dichotomous data as *n* (%). The effect of ERP on covariables was tested by linear regression for continuous variables or logistic regression for dichotomous variables.

a Total cholesterol∶HDL ratio indicative for hypercholesterinaemia.

b Arterial hypertension was defined as systolic blood pressure ≥140 mmHg or diastolic blood pressure ≥90 mmHg or intake of antihypertensive medication.

c Nicotine abuse was defined as past or current smoking.

d Congestive heart failure, myocardial infarction, and diabetes mellitus were based on patients' reports.

e QT interval was corrected according to the Framingham formula: QTc = QT−0.154×(RR−1,000).

*p≤0.05 in bold font.

At baseline, cardiovascular risk factors were significantly more prevalent in deceased individuals than in survivors.

### Prevalence of ERP

**Figure 1:**
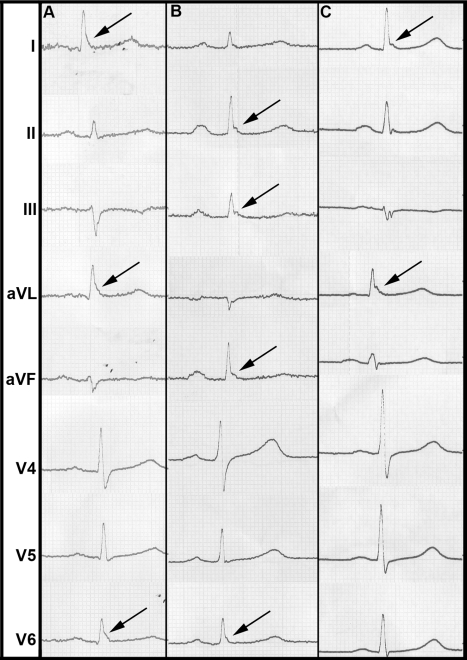
Representative examples of ERP from our study population. (A) shows a slurring morphology, whereas a notching morphology predominates in (B) and (C). Arrows point to leads where ERP can be identified most clearly.

In our study population, the overall prevalence of ERP was 13.1%. ERP was more common in men. It was more prevalent in individuals deceased from cardiac causes. ERP was found predominantly in antero-lateral leads (4.4%) or inferior leads (7.6%) (Table 2). In 1.0%, ERP was present in a combination of antero-lateral and inferior leads. A slurring or notching morphology was present in 9.5% and 3.5%, respectively. These proportions remained stable among sexes. Representative examples of ERP in our study population are shown in Figure 1.

**Table 2:** ERP prevalence and mortality.

ERP Prevalence *n* (%)	*n* Study Population (%)	*n* Death from Cardiac Causes (%)	*n* Death from Any Cause (%)
**Total ** ***n***	6,213	511	1,496
**Overall**	812 (13.1)	89 (17.4)	244 (16.3)
**Antero-lateral leads**	275 (4.4)	25 (4.9)	78 (5.2)
**Inferior leads**	474 (7.6)	58 (11.4)	149 (10.0)
**Combined antero-lateral and inferior leads**	63 (1.0)	6 (1.2)	17 (1.1)
**Slurring morphology**	590 (9.5)	58 (11.4)	161 (10.8)
**Notching morphology**	219 (3.5)	31 (6.1)	83 (5.6)
**Men**	439 (7.1)	60 (11.7)	160 (10.7)
**Women**	372 (6.0)	29 (5.7)	84 (5.6)
**35–54 y**	422 (11.9)	20 (19.0)	57 (15.4)
**55–64 y**	277 (14.3)	51 (20.8)	120 (18.0)
**65–74 y**	114 (15.6)	18 (11.2)	67 (14.6)

Data are displayed in absolute numbers (with percent in parentheses) and present the prevalence of ERP in the study population and in individuals deceased from cardiac or any causes.

### Association of ERP with Cardiac and All-Cause Mortality

Follow-up information was available for a mean duration of 18.9 y (0.03–23.2 y). During this period, 511 individuals died from cardiac causes, of which 345 (67.5%) were of male sex and 89 (17.4%) presented with ERP. A total of 1,496 participants deceased of any cause, of which 932 (62.3%) were male and 244 (16.3%) presented with ERP (Table 2).

We detected a robust relationship between ERP and cardiac mortality. ERP-attributable effects on mortality showed strong age-dependence. Accounting for age-dependence by incorporating an interaction term between ERP and age (ERP × age), we found associations between ERP and cardiac mortality that persistently remained significant after adjusting for age, sex, and survey, or additional clinical risk factors. This effect faded with increasing age. The strongest effects reached an age-dependent hazard ratio (HR) of up to 3.44, which was reduced per additional year of age (Tables 3 and S1).

**Table 3:** Association of ERP with cardiac mortality.

Study Population	Substrata	ERP in any Localization		ERP in Inferior Localization	
		HR (95% CI)	*p*-Value	HR (95% CI)	*p*-Value
**All**					
**Main effect**	**ERP**	3.44 (1.52–7.80)	0.003	3.71 (1.44–9.53)	0.007
	**ERP × age**	0.95 (0.92–0.99)	0.005	0.96 (0.92–1.00)	0.049
**Age-strata**	**35–54 y**	1.96 (1.05–3.68)	0.035	3.15 (1.58–6.28)	0.001
	**55–64 y**	1.12 (0.70–1.78)	0.63	1.33 (0.78–2.27)	0.29
	**65–74 y**	0.59 (0.25–1.44)	0.25	1.18 (0.48–2.92)	0.72
**Women**					
**Main effect**	**ERP**	5.97 (0.85–42.04)	0.073	1.58 (0.14–17.42)	0.71
	**ERP × age**	0.93 (0.86–1.00)	0.56	0.99 (0.90–1.09)	0.91
**Age-strata**	**35–54 y**	1.25 (0.34–4.58)	0.73	1.48 (0.30–7.29)	0.63
	**55–64 y**	0.99 (0.39–2.50)	0.99	1.80 (0.57–5.63)	0.32
	**65–74 y**	0.63 (0.15–2.72)	0.54	0.77 (0.10–6.14)	0.81
**Men**					
**Main effect**	**ERP**	2.69 (1.10–6.60)	0.030	4.32 (1.59–11.68)	0.004
	**ERP × age**	0.96 (0.93–1.00)	0.058	0.96 (0.92–1.00)	0.039
**Age-strata**	**35–54 y**	2.65 (1.21–5.83)	0.015	4.27 (1.90–9.61)	<0.001
	**55–64 y**	1.16 (0.67–2.02)	0.60	1.28 (0.67–2.42)	0.45
	**65–74 y**	0.67 (0.21–2.08)	0.49	0.77 (0.10–6.14)	0.81

Association of ERP with cardiac mortality is displayed for both ERP and for an ERP localization restricted to inferior leads. Results are shown for the entire study population, and separated for women and men. Results for the main effect are derived from a weighted Cox-proportional hazards model-based pooled analysis of the entire study population, incorporating an ERP-age interaction term (ERP × age) to account for age-dependence of ERP. Results for three different age-strata are shown. All calculations are adjusted for sex, age, and survey and for the following clinical covariables: body mass index, total cholesterol/HDL cholesterol ratio, arterial hypertension, nicotine abuse, congestive heart failure, prior myocardial infarction, diabetes mellitus, heart rate, and QTc. In case of age- and/or sex-stratified analyses, no further adjustment was performed for the respective variables.

**Figure 2:**
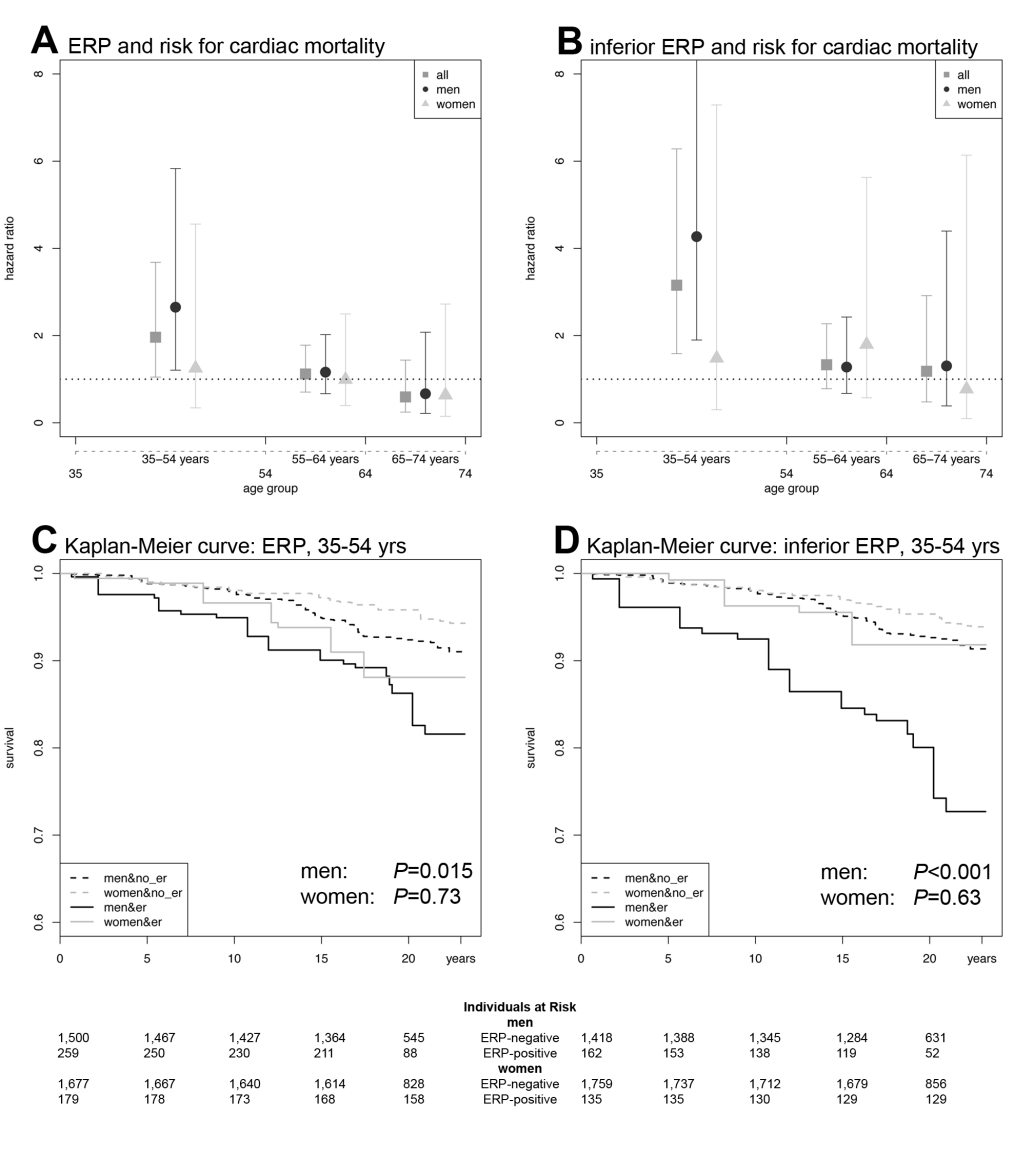
ERP effects on cardiac mortality. (A) HRs and CIs for ERP in any localization. (B) HRs and CIs for the analysis restricted to ERP in an inferior localization. In both (A) and (B), the symbols illustrate the effect size as derived from an age-stratified analysis in younger (35–54 y), middle-aged (55–64 y), and older (65–74 y) participants. Comparing (A) and (B), the ERP-attributable effect is more pronounced if localized in inferior leads. The lower panels show Kaplan-Meier curves for cardiac mortality depending on the presence of ERP and sex in the subgroup of younger individuals (35–54 y). (C) Kaplan-Meier curves for ERP in any localization. (D) Kaplan-Meier curves for ERP in an inferior localization. Men with ERP show the highest cumulative hazard. The effect of ERP on cardiac mortality is stronger in men and outweighs the sex-attributable effect. ERP-attributable effects tend to start earlier in men than in women. Again, effects are stronger, restricting the analysis to an inferior localization of ERP. *p*-Values in (C and D) were derived by a weighted Cox proportional hazards model. The numbers of individuals at risk are listed below each Kaplan-Meier plot.

In age-stratified analyses, in the lowest age-group (35–54 y), we found a persistent and hazardous effect of ERP on cardiac mortality for both sexes (HR 1.96, 95% confidence interval [CI] 1.05–3.68, *p* = 0.035) and males only (HR 2.65, 95% CI 1.21–5.83, *p* = 0.015). Effects were less pronounced in women, where results failed to reach statistical significance (Figure 2A, 2C; Tables 3 and S1). In middle-aged participants (55–64 y), ERP appeared to have no relevant effect on mortality (Figure 2A; Tables 3 and S1).

When stratifying for localization of ERP, we found that an inferior distribution conferred the strongest risk for cardiac mortality in an interaction analysis with age, reaching an age-dependent HR of 3.71. In the age-stratified analysis of 35–54-year-old participants, the HR was 3.15 (95% CI 1.58–6.28, *p* = 0.001) for both sexes and 4.27 (95% CI 1.90–9.61, *p*<0.001) for males only (Figure 2B, 2D; Tables 3 and S1). No relevant effect was determined for antero-lateral distributions of ERP (unpublished data).

The effect of ERP on all-cause mortality was generally weaker, but followed the same pattern as compared to the effect on cardiac mortality. For ERP in any localization, significant associations were observed in an age-dependent analysis of both sexes and in the age-group of 35–54-y-old males. Restricting the analysis to ERP localized in inferior leads, we found significant findings in both an age-dependent and age-stratified model for both sexes combined and males only. Detailed results on all-cause mortality can be derived from Tables 4 and S2.

**Table 4:** Association of ERP with all-cause mortality.

Study Population	Substrata	ERP in any Localization		ERP in Inferior Localization	
		HR (95% CI)	*p*-Value	HR (95% CI)	*p*-Value
**All**					
**Main effect**	**ERP**	1.87 (1.03–3.37)	0.038	2.17 (1.11–4.23)	0.023
	**ERP × age**	0.97 (0.95–1.00)	0.041	0.98 (0.95–1.01)	0.11
**Age-strata**	**35–54 y**	1.39 (0.95–2.03)	0.095	1.80 (1.15–2.81)	0.010
	**55–64 y**	1.00 (0.71–1.40)	0.98	1.18 (0.79–1.77)	0.41
	**65–74 y**	0.77 (0.41–1.45)	0.42	0.13 (0.58–2.20)	0.71
**Women**					
**Main effect**	**ERP**	2.05 (0.62–6.82)	0.24	1.24 (0.30–5.17)	0.77
	**ERP × age**	0.96 (0.91–1.01)	0.12	0.99 (0.93–1.05)	0.63
**Age-strata**	**35–54 y**	0.97 (0.49–1.91)	0.92	0.94 (0.42–2.08)	0.88
	**55–64 y**	0.74 (0.37–1.48)	0.40	1.00 (0.44–2.28)	1.00
	**65–74 y**	0.60 (0.23–1.56)	0.30	0.51 (0.16–1.69)	0.27
**Men**					
**Main effect**	**ERP**	1.67 (0.85–3.30)	0.14	2.62 (1.24–5.57)	0.012
	**ERP × age**	0.98 (0.95–1.01)	0.26	0.97 (0.94–1.01)	0.15
**Age-strata**	**35–54 y**	1.88 (1.15–3.08)	0.012	2.80 (1.61–4.88)	<0.001
	**55–64 y**	1.11 (0.74–1.67)	0.61	1.35 (0.83–2.20)	0.23
	**65–74 y**	1.02 (0.42–2.48)	0.96	1.55 (0.58–4.13)	0.29

Association of ERP with all-cause mortality is displayed for both ERP and for an ERP localization restricted to inferior leads. Results are shown for the entire study population, and separated for women and men. Results for the main effect are derived from a weighted Cox-proportional hazards model-based pooled analysis of the entire study population, incorporating an ERP-age interaction term (ERP × age) to account for age-dependence of ERP. Results for three different age-strata are shown. All calculations are adjusted for sex, age, and survey and for the following clinical covariables: body mass index, total cholesterol/HDL cholesterol ratio, arterial hypertension, nicotine abuse, congestive heart failure, prior myocardial infarction, diabetes mellitus, heart rate, and QTc. In case of age- and/or sex-stratified analyses, no further adjustment was performed for the respective variables.

## Discussion

In our study, we determined the prevalence of ERP to be 13.1% in the overall sample. Using long follow-up, we were able to demonstrate a strong association between ERP and cardiac mortality. This association was strongest for the presence of ERP in inferior leads of the ECG and restricted to men between 35–54 y of age. Here, the HR was 4.32. To a lesser extent, we also detected an association between ERP and all-cause mortality.

This study determined the prevalence of ERP in a large population-based sample. The overall prevalence of ERP was 13.1%, and men were more frequently affected than women. This finding contrasts previous publications reporting a prevalence between 1%–5% [3]–[5]. These studies were derived from large patient or volunteer samples of 60,000 and 73,000 individuals, respectively, and recently from a population-based study in Finland where the prevalence of ERP was 5.8% [8]. This latter cohort was recruited from various sites across Finland and thus might represent individuals of Northern-European descent only. Our population, however, is of Central-European descent and was homogeneously recruited from a well-defined single region of Southern Germany. Another Western-European population in France evaluated females only and reported an ERP prevalence of 17.2% [21].

Interestingly, in 1936 one of the first, though not population-based, studies reporting the prevalence of ERP found 25% and 16% in men and women, respectively [2]. Importantly, definition of ERP was heterogeneous across studies. Our results strictly relied on the definition by Haïssaguerre and colleagues [7], and support the clinical experience that ERP is indeed a common ECG signature in every-day practice.

Our study found an association of ERP with both cardiac and all-cause mortality. ERP was most pronounced in younger individuals, particularly in males, where the ERP-associated cardiac risk was found to be increased 2–4-fold. For many years ERP was considered a benign phenomenon. However, this judgment was seriously questioned by both (1) a study, in which ERP was found to be a clinically relevant ECG signature, and possibly a marker of SCD, in the evaluation of unexplained syncope or idiopathic ventricular fibrillation in patients without structural heart disease [7], and (2) the recent report associating ERP with cardiovascular mortality in a Finnish community-based population [8]. A difference between the initial clinical report, the recent population-based study, and our study are the investigated populations. While Haïssaguerre and coworkers presented results of selected SCD-survivors, and the initial population-based study reported on a Northern-European population, we report here on a large community-based cohort of individuals of Central-European origin. However, all of these recent studies applied the same definition of ERP, making the results comparable. Furthermore, in the Finnish study [8] and in our investigation, the ECGs were recorded prospectively, before the occurrence of events, and in both, ERP was found more frequently in males. Our study provides evidence from the general population supporting the hypothesis that ERP is a marker for increased risk for cardiac mortality. These findings support the results by Tikkanen and colleagues, where a slightly increased risk for cardiovascular mortality was shown in an unstratified sample applying a comparable definition of ERP [8]. A hazardous effect of ERP is further suggested by the fact that an association can still be detected looking at all-cause mortality, although the effect size and association strength are much more pronounced when investigating cardiac mortality only.

In addition to the previous reports, our study further refines the association of ERP with cardiac mortality, demonstrating an association between ERP and cardiac mortality predominantly in younger individuals. SCD occurs in approximately 50% of all cardiovascular deaths [10]. Coronary artery disease is the main underlying disease primarily in the elderly, while in younger individuals SCD without underlying structural heart disease is more common [10]. For these reasons, a pathophysiologic link between ERP and malignant arrhythmias seems plausible, and would suggest that ERP-attributable death occurred without underlying structural defect. However, the cause of death in our study was determined from death certificates only. Thus, there is a possible bias towards common causes of death. Yet, in the context of structural heart disease, ERP could modify the risk for malignant arrhythmias. Additionally, the attenuated association between ERP and cardiovascular mortality in older age groups might be attributed to an increased prevalence and relevance of classical risk factors. Our results were therefore adjusted for a variety of cardiovascular risk factors, and the association between ERP and cardiac mortality became much more pronounced thereafter.

A sex-stratified analysis revealed a particularly strong association of ERP with cardiac mortality in males. Conflicting data exist as to the predominance of sex in SCD and sudden unexplained death (SUD). Against the general notion that SUD is more common in men—autopsy series reported 63%–68% males [22],[23]—the Edwards Registry of Cardiovascular Death found a clear female predominance for SUD (32% and 24% for women and men, respectively). After detailed review, the rates of SCD in these individuals were 50% in women versus 24% in men [24]. A study in female army recruits found SUD to be attributable to 53% of sudden deaths [25]. This trend towards women holds true also in the context of ischemic events. Dekker and colleagues showed that women tended to present more often with ventricular fibrillation in the context of a first myocardial infarction than men (21.8% versus 16.7%, *p* = 0.084) [26]. Our data, as well as the previous report by Tikkanen and colleagues [8], demonstrate a higher prevalence of ERP in men, which is associated with higher cardiac and all-cause mortality. This result suggests that, among all causes of SCD, ERP might be a risk factor for SCD predominantly in males.

The potentially hazardous nature of ERP is also supported by experimental, electrophysiological data [6]. A transmural heterogeneity, primarily generated by the I_to_-channel, underlies the presence of ERP on the surface ECG. This transmural gradient is caused by an epicardial but not endocardial depression of the action potential plateau. Compared to arrhythmogenic entities like the Brugada syndrome, in ERP this gradient is relatively weak, but can be aggravated by certain triggers like drugs, vagal tone, or electrolyte shifts. This tendency might lead to the interpretation that ERP is not a malignant condition per se, but rather represents a susceptibility marker for malignant arrhythmias, possibly in the context of a triggering event. Further research is warranted to identify potential triggers. As seen for a number of other ECG signatures, for example QT- or PR-interval [27]–[29], among other factors, there might also be a heritable component, explaining part of the variable occurrence of ERP in the population or the degree of arrhythmogenicity.

With regard to the clinical impact of ERP, a careful interpretation is required. Younger males (35–54 y) in our study appear to bear the highest risk. However, the maximum risk attributed to ERP might be overestimated because of low incidence of cardiac deaths in the younger age groups. ERP is associated with a 2–4-fold increased risk, which parallels the findings for the risk of cardiovascular mortality attributed to established ECG parameters. In the Rotterdam Study, a prolonged QTc interval conferred a HR between 1.3 and 2.4 for men and women, respectively [30]. In a study analyzing the risk of SCD, an abnormally long QTc (≥450 ms in men; ≥470 ms in women) was associated with a 2.6-fold increased risk in men and 2.5-fold in women, while younger participants appeared to be at higher risk [31]. For resting heart rate >75 beats/min, the Paris Prospective Study reported a maximum HR of 3.5 for SCD [32]. Both QT prolongation and resting heart rate found their way into clinical routine, as the ECG is a diagnostic tool widely used in clinical practice. Yet, the impact of QTc or heart rate is still low in otherwise asymptomatic individuals, unless extreme values are detected, family history of SCD is positive, or symptoms occur. The conclusion might be drawn that the detection of ERP on a routine ECG screen in an asymptomatic individual with a negative family history for SCD does not need to be followed up by a work-up or even prophylactic therapy. In this context, in accordance with Tikannen and colleagues, we propose to pay special attention to individuals with ERP in inferior leads. In addition there is evidence that the currently applied threshold for ERP (≥0.1 mV) appears to be too low [8]. We aimed at substantiating this notion; however, our study sample was not adequately powered to reliably perform an analysis with an elevated ERP threshold.

### Strengths and Limitations

A major strength of our study is the use of the MONICA/KORA cohort being characterized by long follow-up and detailed risk factor and ECG information. The case-cohort design provides an efficient and unbiased sample of the entire cohort [13],[14]. Likewise, the systematic and blinded fashion of the ECG analysis can be considered a strong point.

However, several limitations merit discussion. A weakness is the ascertainment of death by death certificates only. As discussed above, a reliable differentiation can be made between cardiovascular and noncardiovascular causes. However, within cardiovascular causes, a more accurate differentiation is often not feasible. We aimed to enrich cases of cardiac death within the spectrum of cardiovascular causes of death, but it will require further studies to narrow down the real underlying causes of death to foster the hypothesis of a presumably arrhythmic death due to ERP.

Another limitation is the strictly epidemiologic nature of our investigation. Consequently we cannot further clarify the pathophysiologic and pathogenetic circumstances underlying ERP. Inherent to our study design, we cannot comment on the influence of ERP outside the age range of 35–74 y. Further research is warranted to elucidate the impact of ERP in younger individuals. Repetitive recordings of ECGs in the same individuals are currently not available. We thus cannot comment on whether ERP is a permanent or transient ECG pattern. However, Tikkanen and colleagues reported persistence of ERP in >80% of repeated ECG recordings [8].

In conclusion, we report a high prevalence of ERP in the general population. Moreover, we found a robust association of ERP with cardiac, and to a lesser degree, all-cause mortality in participants between ages 35–54 y. The ERP-associated risk was in the range of that conferred by prolonged QTc or elevated resting heart rate in the general population. There is evidence that a localization of ERP in inferior leads is associated with the highest risk.

## Supporting Information

Table S1 Association of ERP with cardiac mortality (age-, sex-, and survey-adjusted model).
(0.07 MB DOC)

Table S2 Association of ERP with all-cause mortality (age-, sex-, and survey-adjusted model).
(0.07 MB DOC)

## Abbreviations

CI: confidence interval
ECG: electrocardiogram
ERP: early repolarization pattern
HDL: high-density lipoprotein
HR: hazard ratio
ICD-9: 9th revision of the International Classification of Disease
KORA: Cooperative Health Research in the Region of Augsburg
MONICA: Monitoring of cardiovascular diseases and conditions
SCD: sudden cardiac death
SUD: sudden unexplained death
