# Supplementary material for: Association of Early Repolarization Pattern on ECG with Risk of Cardiac and All-Cause Mortality: A Population-Based Prospective Cohort Study (MONICA/KORA)
Source: PLoS Med. 2010 Jul 27;7(7):e1000314. doi: 10.1371/journal.pmed.1000314 (PMC2910598; doi:10.1371/journal.pmed.1000314)
Supplement: Table S1 — Association of ERP with cardiac mortality (age-, sex-, and survey-adjusted model). (0.07 MB DOC) [file pmed.1000314.s001.doc]

**Table S1: Association of ERP with Cardiac Mortality (age, sex and survey adjusted model).**

|  |  | **ERP in any localization** |  |  | **ERP in inferior localization** |  |
| --- | --- | --- | --- | --- | --- | --- |
|  |  | Hazard ratio |  |  | Hazard ratio |  |
|  |  | (95% confidence interval) | *p-value* |  | (95% confidence interval) | *p-value* |
| **All** |  |  |  |  |  |  |
| Main effect | ERP | 2.88 (1.30 – 6.39) | **0.009** |  | 3.28 (1.34 – 8.04) | **0.010** |
|  | ERP*age | 0.96 (0.93 – 1.00) | **0.027** |  | 0.97 (0.93 – 1.00) | 0.084 |
| Age-strata | 35-54 years | 1.66 (0.97 – 2.83) | 0.063 |  | 2.19 (1.21 – 3.95) | **0.010** |
|  | 55-64 years | 1.36 (0.91 – 2.03) | 0.13 |  | 1.67 (1.04 – 2.68) | **0.034** |
|  | 65-74 years | 0.63 (0.32 – 1.24) | 0.19 |  | 0.86 (0.40 – 1.84) | 0.70 |
| **Women** |  |  |  |  |  |  |
| Main effect | ERP | 4.99 (0.96 – 25.78) | 0.055 |  | 2.24 (0.33 – 15.17) | 0.41 |
|  | ERP*age | 0.94 (0.88 – 1.01) | 0.077 |  | 0.98 (0.91 – 1-06) | 0.59 |
| Age-strata | 35-54 years | 1.66 (0.60 – 4.58) | 0.33 |  | 1.33 (0.39 – 4.56) | 0.65 |
|  | 55-64 years | 1.43 (0.71 – 2.89) | 0.32 |  | 2.25 (0.96 – 5.31) | 0.63 |
|  | 65-74 years | 0.49 (0.16 – 1.50) | 0.21 |  | 0.52 (0.12 – 2.17) | 0.37 |
| **Men** |  |  |  |  |  |  |
| Main effect | ERP | 2.33 (0.93 – 5.86) | 0.071 |  | 3.50 (1.28 – 9.58) | **0.015** |
|  | ERP*age | 0.97 (0.94 – 1.01) | 0.16 |  | 0.97 (0.93 – 1.01) | 0.12 |
| Age-strata | 35-54 years | 1.73 (0.91 – 3.29) | 0.096 |  | 2.59 (1.29 – 5.17) | **0.007** |
|  | 55-64 years | 1.30 (0.80 – 2.11) | 0.30 |  | 1.51 (0.85 – 2.70) | 0.16 |
|  | 65-74 years | 0.73 (0.31 – 1.71) | 0.47 |  | 1.04 (0.42 – 2.61) | 0.93 |

Association of ERP with cardiac mortality is displayed for both ERP and for an ERP localization restricted to inferior leads. Results are shown for the entire study population, and separated for women and men. Results for the main effect are derived from a weighted Cox-proportional hazards model based pooled analysis of the entire cohort incorporating an ERP-age interaction term (ERP*age) to account for age-dependence of ERP. Results for three different age-strata are shown. Calculations are adjusted for age, sex and survey. In case of age- and / or sex-stratified analyses, no further adjustment was performed for the respective variables.
